# Clinical outcomes for olfactory neuroblastoma

**DOI:** 10.3389/fonc.2024.1329572

**Published:** 2024-05-02

**Authors:** Akira Nakazono, Hiroaki Motegi, Masanobu Suzuki, Yuji Nakamaru, Shigeru Yamaguchi, Yukitomo Ishi, Satoshi Kano, Nayuta Tsushima, Aya Honma, Takayoshi Suzuki, Shogo Kimura, Seijiro Hamada, Jun Taguchi, Yasushi Shimizu, Takashi Mori, Koichi Yasuda, Hidefumi Aoyama, Ichiro Kinoshita, Miki Fujimura, Akihiro Homma

**Affiliations:** ^1^ Department of Otolaryngology-Head and Neck Surgery, Faculty of Medicine and Graduate School of Medicine, Hokkaido University, Sapporo, Japan; ^2^ Department of Neurosurgery, Faculty of Medicine and Graduate School of Medicine, Hokkaido University, Sapporo, Japan; ^3^ Department of Medical Oncology, Faculty of Medicine and Graduate School of Medicine, Hokkaido University, Sapporo, Japan; ^4^ Department of Radiation Oncology, Faculty of Medicine and Graduate School of Medicine, Hokkaido University, Sapporo, Japan

**Keywords:** olfactory neuroblastoma, esthesioneuroblastoma, endoscopic skull base surgery, craniotomy, induction chemotherapy, postoperative radiotherapy

## Abstract

**Background:**

Olfactory neuroblastoma (ONB) is a rare malignant tumor arising from the olfactory neuroepithelium. The standard of care for ONB is surgical resection; however, detailed treatment protocols vary by institution. Our treatment protocol consists of endoscopic skull base surgery (ESBS) for endoscopically resectable cases and induction chemotherapy followed by craniotomy combined with ESBS for locally advanced cases, with postoperative radiotherapy performed for all cases. Chemoradiotherapy (CRT) is performed in unresectable cases. In this study, we evaluate our treatment protocol and outcomes for ONB.

**Methods:**

A retrospective review of patients with ONB was conducted. Outcomes included survival outcomes and perioperative data.

**Results:**

Fifteen patients (53.6%) underwent ESBS, 12 (42.9%) underwent craniotomy combined with ESBS, and 1 (3.6%) received CRT. The 5- and 10-year overall survival rates for all patients were 92.9% and 82.5%, respectively, with a median follow-up period of 81 months. The 5- and 10-year disease-free survival rates were 77.3% and 70.3%, respectively, and the 5- and 10-year local control rates were 88.2% and 80.2%, respectively. Patients undergoing ESBS demonstrated a significantly shorter operating time, period from operation to ambulation, hospitalization period, and less blood loss than those undergoing craniotomy combined with ESBS.

**Conclusion:**

Our treatment protocol was found to afford favorable outcomes. Patients who underwent endoscopic resection showed lower complication rates and better perioperative data than those who underwent craniotomy combined with ESBS. With appropriate case selection, ESBS is considered a useful approach for ONB.

## Introduction

Olfactory neuroblastoma (ONB) is a rare malignant tumor arising from the olfactory neuroepithelium. Since it was first described by Berger et al. in 1924 (1), over 1000 cases have been reported. However, it remains a rare neoplasm, representing only about 3-6% of sinonasal malignancies (2). Cervical lymph node metastasis is seen in around 10-15% of cases, and distant metastasis in less than 10% (3–6).

The standard of care for ONBs is considered to be complete resection of the tumor, followed by postoperative radiotherapy (7, 8). Although craniofacial resection has traditionally been performed to resect ONBs, recent advances in endoscopic skull base surgery (ESBS) have enabled complete resection of the tumor by ESBS alone (8–10). Currently, it is reported that 32.5-65% of cases are resected by ESBS (11, 12).

On the other hand, tumors extending into areas that cannot be managed via an endonasal approach (i.e., skin, optic nerve, lateral orbital extension, extensive dural and brain invasion, and carotid artery) are not indicated for ESBS. Such cases should be managed by craniotomy with/without ESBS (8, 13).

In this study, we report on the course of treatment of ONB over a 15-year period at our hospital. We also report the prognosis and perioperative course in cases treated with endoscopic resection and open surgery.

## Patients and methods

### Patients

We retrospectively reviewed patients with ONB treated between 2003 and 2023 at Hokkaido University Hospital. All patients were evaluated by the Cancer Board, consisting of rhinologists, head and neck surgeons, neurosurgeons, radiation oncologists, diagnostic radiologists, and medical oncologists. Each case was classified according to the modified Kadish and Dulguerov staging systems. The staging for each case was determined based on physical examination, computed tomography (CT), and magnetic resonance imaging (MRI). We also reviewed medical records for patient characteristics, perioperative data, and histochemical findings for Hyams grade and surgical margin evaluation.

### Treatment strategies

All cases were discussed by the Cancer Board. Until 2008, all cases were treated with craniofacial resection; however, after 2008, ESBS was performed in cases without invasion to the lateral brain parenchyma or a wide range of dura mater beyond the medial orbital lines and the frontal baselines. In more advanced cases, induction chemotherapy (IC) was performed to reduce tumor volume, followed by craniotomy combined with ESBS. Intraoperative frozen section diagnosis confirmed complete resection of the tumor (samples for frozen section diagnosis were obtained from the nasal mucosa, circumferential dura mater, and olfactory bulb). The skull base was reconstructed by fascia, fat, and a nasal septal flap in ESBS, or by a pericranial flap in craniotomy combined with ESBS. In principle, postoperative radiotherapy (until 2004 65Gy/26fr, thereafter 60Gy/30fr) was performed in all cases, regardless of surgical approach or pathological margin status. In surgical cases, the gross tumor volume (GTV) was defined as residual tumor if residual tumor was present, or no GTV if no residual tumor was present. In nonoperative cases, GTV was defined as tumor seen on CT or MRI. The clinical target volume (CTV) consisted of the preoperative tumor with an expansion of 5-10 mm in all directions in principle. The entire nasal cavity, ethmoid sinus, sphenoid sinus, and maxillary sinus were also set for CTV. Possible tumor invasion of brain parenchyma, nasopharynx, or frontal sinus was included in CTV, while extrabony and orbits were excluded. If the primary tumor was confined to one side, the healthy maxillary sinus was also excluded from CTV. Prophylactic irradiation of the neck was not performed, but in one case with preoperative evidence of cervical lymph node metastasis, bilateral cervical lymph node areas and ipsilateral supraclavicular lymph node area on the affected side were set as CTV.

### Statistical analysis

The probabilities of overall survival (OS), disease-free survival (DFS), and local control (LC) were analyzed by the Kaplan-Meier method. OS was defined as the interval between the beginning of primary treatment and the date of death or the last visit. DFS was defined as the interval between the beginning of primary treatment and the date of death or cancer recurrence confirmed in any site or the date of the last visit. LC was defined as the interval between the beginning of primary treatment and the date of cancer recurrence confirmed in a local site or the date of the last visit. The log-rank test was used for survival comparisons. All averages are listed as average ± standard error. Perioperative data were compared using the Mann-Whitney U test, and all tests were two-sided. A p-value of <0.05 was considered significant. For comparison among three or four groups, the Bonferroni correction was applied and p-values of less than 0.017 for three groups (i.e., 0.05/3 = 0.017), and 0.0083 for four groups (i.e., 0.05/6 = 0.0083) were considered statistically significant, respectively.

All statistical analyses were performed with EZR (14) (Saitama Medical Center, Jichi Medical University, Saitama, Japan), which is a graphical user interface for R (The R Foundation for Statistical Computing, Vienna Austria).

## Results

### Patient characteristics

Twenty eight patients were retrospectively reviewed based on their medical records. The patients consisted of 10 females and 18 males, with a median age of 54 years (range 13-74 years, average 52.6 ± 2.8 years). The median follow-up period was 81 months (range 8-226 months, average 82.1 ± 10.2 months). With regard to Hyams grading, one patient was diagnosed with grade I, 18 with II, 7 with III, and 2 with IV disease (**Table 1**). Under the modified Kadish staging system, three patients were diagnosed with stage A, 7 with B, 16 with C, and 2 with D disease. Based on the Dulguerov staging system, six patients were diagnosed with T1, 3 with T2, 3 with T3, and 16 with T4 disease. Two patients (Dulguerov T2 and T4) had lymph node metastasis, and no patients had distant metastasis.

**Table 1:** Patient characteristics and treatment modalities.

Sex	No.	(%)
Male	18	(64.3)
Female	10	(35.7)
Age	median	(range)
	54	(13-74)
Observation period	median	(range)
	81	(8-226)
Hyams grade	No.	(%)
I	1	(3.6)
II	18	(64.3)
III	7	(25)
IV	2	(7.1)
Modified Kadish stage	No.	(%)
A	3	(10.7)
B	7	(25)
C	16	(57.1)
D	2	(7.1)
Dulguerov T stage	No.	(%)
T1	6	(21.4)
T2	3	(10.7)
T3	3	(10.7)
T4	16	(57.1)
Dulguerov N stage	No.	(%)
N0	26	(92.9)
N1	2	(7.1)
Induction chemotherapy	No.	(%)
Yes	14	(50)
No	14	(50)
Treatment	No.	(%)
ESBS	15	(53.6)
Craniotomy combined with ESBS	12	(42.9)
CRT	1	(3.6)

ESBS, endoscopic skull base surgery; CRT, chemoradiotherapy.

### Treatment and clinical course

Of the 28 patients, 27 patients were treated with surgery and 1 with CRT. Of the 27 patients who underwent surgical treatment, 12 patients received craniofacial resection-assisted endoscopy, and 15 patients received ESBS. Induction chemotherapy was performed for 1 patient who underwent chemoradiotherapy, eleven patients who underwent craniofacial resection-assisted endoscopy and two patients who underwent ESBS. Eleven patients were treated with the ICE regimen (ifosfamide 900 mg/m2, cisplatin 20 mg/m2, and etoposide 60 mg/m2), one patient was treated with cisplatin (80 mg/m2), one patient was treated with cisplatin (80 mg/m2) and etoposide (100 mg/m2), and one patient was treated with the CADO-CVP regimen (cyclophosphamide 250 mg/m2, Adriamycin 50 mg/m2, vincristine 1.2 mg/m2, cisplatin 25 mg/m2, etoposide 80 mg/m2). Of the 14 cases, 4 demonstrated a partial response (PR) and 10 had stable disease (SD).

Treatment modalities according to modified Kadish and Dulguerov staging systems are shown in **Table 2**.

**Table 2:** Treatment modalities in each staging system.

Staging system No. (%)				Treatment No. (%)					
				ESBS		Craniotomy combined with ESBS		CRT	
modified Kadish	A	3	(10.7)	3	(10.7)	0	(0)	0	(0)
	B	7	(25)	6	(21.4)	1	(3.6)	0	(0)
	C	16	(57.1)	5	(17.9)	11	(39.3)	0	(0)
	D	2	(7.1)	1	(3.6)	0	(0)	1	(3.6)
Dulguerov	T1	6	(21.4)	6	(21.4)	0	(0)	0	(0)
	T2	3	(10.7)	2	(7.1)	1	(3.6)	0	(0)
	T3	3	(10.7)	1	(3.6)	2	(7.1)	0	(0)
	T4	16	(57.1)	6	(21.4)	9	(32.1)	1	(3.6)

ESBS, endoscopic skull base surgery; CRT, chemoradiotherapy.

All patients except one received postoperative radiotherapy regardless of the pathological margin status. Two patients treated before 2004 were irradiated with 65 Gy/26fr and 25 patients treated after 2005 were irradiated with 60 Gy/30fr. Prior to 2013, 8 patients were treated with three-dimensional conformal radiation therapy (3D-CRT), with 19 patients treated with intensity-modulated radiation therapy (IMRT) thereafter. One patient did not receive radiation therapy because the tumor was confined to the nasal cavity, and the resection margins were negative.

In one patient (modified Kadish stage D, Dulguerov T4, and Hyams grade IV), the tumor was huge. As there was no indication for surgery, the patient received CRT (54Gy/27fr with weekly cisplatin 40 mg/m2) after induction chemotherapy.

Six patients experienced recurrence after surgery followed by radiotherapy. Four cases currently show no evidence of disease, and two died of disease at 32 and 65 months. Recurrence patterns and salvage treatments are shown in **Table 3**.

**Table 3:** Cases of recurrence.

Case	Age	Sex	mKadish stage	Dulguerov T stage	Initial treatment	Recurrence (from initial treatment)	Salvage treatment	Outcome (from salvage)
1	66	M	C	T3	IC Craniotomy with ESBS	Dura matter (85 months)	Craniotomy RT (IMRT, 50Gy/25fr)	NED (124 months)
2	44	F	C	T4	IC Craniotomy with ESBS	Brain metastasis (37 months)	RT (3D-CRT, 36Gy/16fr)	DOD (65 months)
3	48	F	C	T4	ESBS	Neck lymph node (8 months)	Neck dissection	NED (44 months)
4	62	M	C	T4	ESBS	Rouviere lymph node (36 months)	RT (proton beam, 65Gy/25fr)	NED (32 months)
5	71	M	C	T4	ESBS	Neck lymph node (20 months)	Neck dissection	NED (48 months)
6	54	F	C	T4	IC ESBS	Dura matter, Neck lymph node (18 months)	RT (IMRT, 35Gy/15fr) Neck dissection	DOD (32 months)

mKadish, modified Kadish.

IC, induction chemotherapy.

ESBS, endoscopic skull base surgery.

RT, radiotherapy.

IMRT, intensity-modulated radiation therapy.

3D-CRT, three-dimensional conformal radiation therapy.

NED, no evidence of disease.

DOD, died of disease.

One patient treated with CRT had meningeal dissemination two months after CRT and died after 3 months.

### Treatment outcomes in ONBs

**Figure 1:**
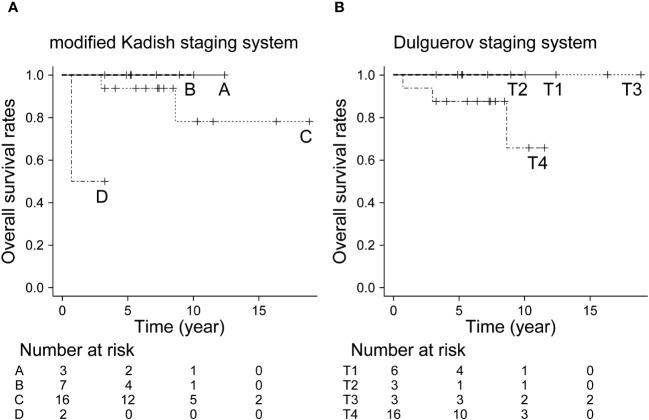
Overall survival rate by staging system. **(A)** Modified Kadish staging system (p<0.05, Bonferroni test), **(B)** Dulguerov staging system (p=0.43, Bonferroni test).

**Figure 2:**
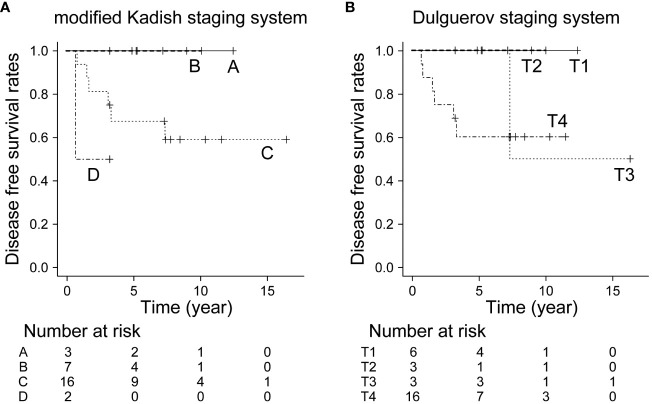
Disease-free survival rate by staging system. **(A)** Modified Kadish staging system (p=0.108, Bonferroni test), **(B)** Dulguerov staging system (p=0.283, Bonferroni test).

**Figure 3:**
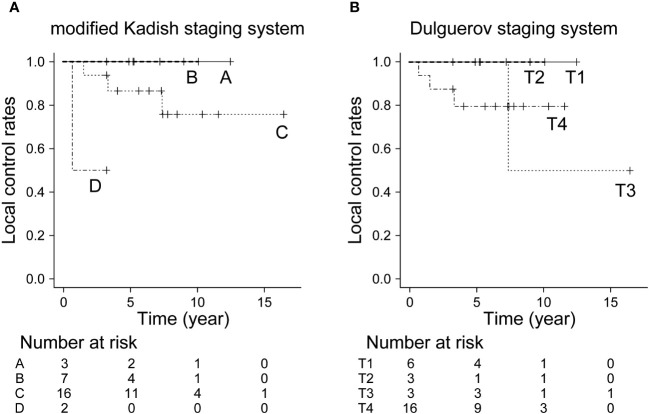
Local control rate by staging system. **(A)** Modified Kadish staging system (p<0.05, Bonferroni test), **(B)** Dulguerov staging system (p=0.599, Bonferroni test).

The 5- and 10-year OS in all patients were 92.9% and 82.5%, respectively. The 10-year OS for modified Kadish staging A, B, C, and D was 100%, 100%, 78.1%, and 69.5%, respectively (p<0.05, **Figure 1A**). The 10-year OS for Dulguerov staging T1, T2, T3, and T4 was 100%, 100%, 100% and 65.6%, respectively (p=0.43, **Figure 1B**). DFS and LCR are shown in **Figures 2** and **3**, respectively.

**Figure 4:**
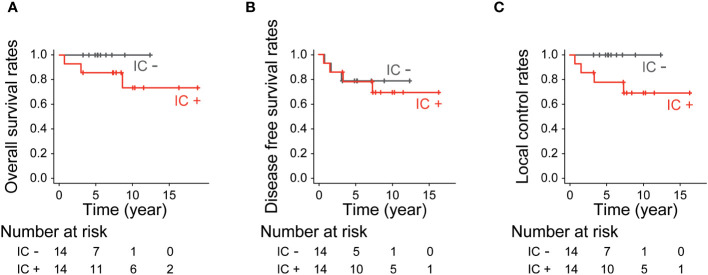
Treatment outcomes by induction chemotherapy. **(A)** Overall survival rates (p=0.13, log-rank test), **(B)** Disease-free survival rates (p=0.95, log-rank test) and **(C)** Local control rates (p=0.081, log-rank test).

The 10-year OS for patients who received and did not receive IC was 73.5% and 100%, respectively (p=0.13, **Figure 4A**). The 10-year DFS for patients who received and did not receive IC was 69.3% and 78.6%, respectively (p=0.95, **Figure 4B**). The 10-year LCR for patients who received and did not receive IC was 69.3% and 100%, respectively (p=0.081, **Figure 4C**).

**Figure 5:**
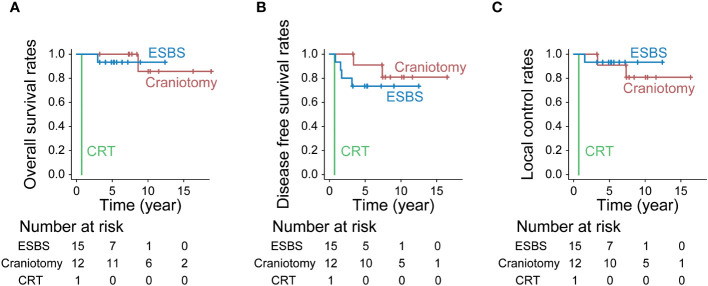
Treatment outcomes by treatment. **(A)** Overall survival rates (p<0.001, Bonferroni test), **(B)** Disease-free survival rates (p<0.001, Bonferroni test) and **(C)** Local control rates (p<0.001, Bonferroni test).

The 10-year OS for ESBS, craniotomy combined with ESBS, and CRT was 93.3%, 85.7%, and 0%, respectively (p<0.001, **Figure 5A**). The 10-year DFS for ESBS, craniotomy combined with ESBS, and CRT was 73.3%, 80.8%, and 0%, respectively (p<0.001, **Figure 5B**). The 10-year LCR for ESBS, craniotomy combined with ESBS, and CRT was 93.3%, 80.8%, and 0%, respectively (p<0.001, **Figure 5C**).

### Operative and perioperative data

**Figure 6:**
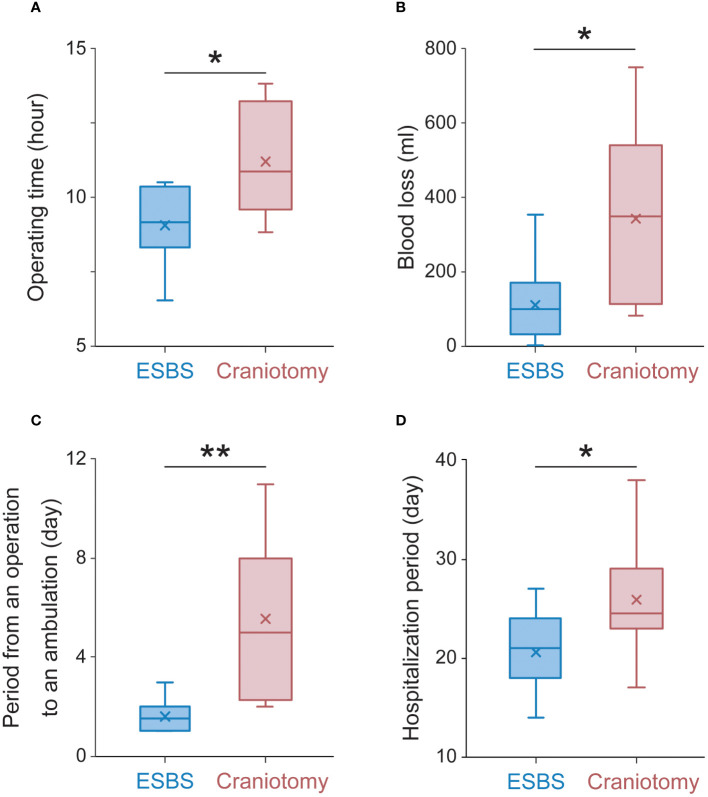
Operative and perioperative data. **(A)** The average operation time, **(B)** the average blood loss, **(C)** the average period from operation to ambulation, and **(D)** the average hospitalization period. The Box plots to show median (horizontal line), mean (cross), upper and lower quartiles (box), maximum and minimum (vertical line). The Mann-Whitney U test was used to determine P values for the indicated comparisons. **p<0.01 *p<0.05.

The median operation time was 9.2 hours (range 6.6-10.5 hours, average 9.1 ± 0.4 hours) in the ESBS group and 10.2 hours (range 8.8-13.8 hours, average 11.2 ± 0.7 hours) in the craniotomy combined with ESBS group (p<0.05, **Figure 6A**). The median blood loss was 113 ml (range 0-355 ml, average 131 ± 35 ml) in the ESBS group and 350 ml (range 80-750 ml, average 343 ± 84 ml) in the craniotomy combined with ESBS group (p<0.05, **Figure 6B**). The median period from operation to ambulation was 1 day (range 1-3 days, average 1.6 ± 0.2 days) in the ESBS group and 5 days (range 2-11 days, average 5.5 ± 1.1 days) in the craniotomy combined with ESBS group (p<0.01, **Figure 6C**), while the median hospitalization period was 21 days (range 14-27 days, average 21 ± 1.3 days) in the ESBS group and 25 days (range 17-38 days, average 25.9 ± 2.2 days) in the craniotomy combined with ESBS group (p<0.05, **Figure 6D**).

### Complications

Four of the 12 patients treated with craniotomy had severe postoperative complications (p<0.05, **Table 4**). One had cerebrospinal fluid (CSF) leakage one month after the craniotomy, which was rescued by endoscopic repair of the skull base using a nasal septal flap. Another had deep vein thrombosis one month after the craniotomy combined with ESBS and was treated with warfarin for 6 months. The remaining two patients treated with craniotomy combined with ESBS and postoperative radiotherapy suffered from necrosis of the frontal bone at 33 and 21 months after surgery. Both patients needed necrotic bone removal and reconstruction of the skull base using a latissimus dorsi flap and local skin flap, respectively.

**Table 4:** Complications associated with the surgical approach.

Complications No. (%)	Treatment				P-value
	ESBS n=15		Craniotomy Combined with ESBS n=12		
**Severe complications**	0	(0)	4	(33.3)	<0.05
Frontal bone necrosis	0	(0)	2	(16.7)	
Cerebrospinal fluid leakage	0	(0)	1	(8.3)	
Deep vein thrombosis	0	(0)	1	(8.3)	

ESBS, endoscopic skull base surgery.

There have been no significant complications to date in patients who underwent ESBS alone.

## Discussion

The standard of care for ONB is considered to be surgery; however, the details of treatment protocols vary from institution to institution. Controversy remains over whether to administer induction chemotherapy or postoperative radiotherapy, as well as whether to perform endoscopic or craniofacial resection. The reasons for the lack of consensus may be due in part to the fact that ONB is a rare disease, making it difficult to conduct adequate clinical trials. Postoperative radiotherapy has been reported to be effective, particularly for cases with locally advanced disease (15). Moreover, the combination of surgery and postoperative radiotherapy has been reported to have more favorable outcomes than surgery alone (16, 17). Prophylactic irradiation of the neck significantly reduced neck recurrence in patients with N0, but did not improve survival rates (18–20). Although we did not provide prophylactic irradiation of the neck, the survival rates were favorable in our case study, as a result.

Currently, the effect of chemotherapy for ONB is unclear. While some reported that chemotherapy might be effective in locally advanced cases (21) and patients with Hyam grade III/IV or Kadish C/D (22, 23), others reported limited efficacy of chemotherapy (24, 25). Early surgical intervention for ONB is important (26), and induction chemotherapy has been reported to be harmful by delaying surgical treatment from the report of 797 cases (27). In our cases, induction chemotherapy is performed in locally advanced cases that require craniotomy combined with ESBS. Of the cases treated with induction chemotherapy, 71.4% (10/14) demonstrated an SD, even though most of them were advanced cases (**Table 5**). It is necessary to consider the therapeutic indications of induction chemotherapy.

**Table 5:** Patient characteristics in patients who received and did not receive induction chemotherapy.

Patient characteristics		Induction chemotherapy				P value
		Yes	n=14	No	n=14	
Sex	No (%)					n.s.
Male		9	(64.3)	9	(64.3)	
Female		5	(35.7)	5	(35.7)	
Age	median (range)					n.s.
		55	(13-74)	45	(23-71)	
Observation period	median (range)					<0.05
		60	(13-148)	101	(8-226)	
Hyams grade	No (%)					n.s.
I		1	(7.1)	0	(0)	
II		11	(78.6)	7	(50)	
III		2	(14.3)	5	(35.7)	
IV		0	(0)	2	(14.3)	
Modified Kadish stage	No (%)					<0.05
A		3	(21.4)	0	(0)	
B		6	(42.9)	1	(7.1)	
C		5	(35.7)	11	(78.6)	
D		0	(0)	2	(14.3)	
Dulguerov T stage	No (%)					n.s.
T1		6	(42.9)	0	(0)	
T2		1	(7.1)	2	(14.3)	
T3		1	(7.1)	2	(14.3)	
T4		6	(42.9)	10	(71.4)	
Dulguerov N stage	No (%)					n.s.
N0		14	(100)	12	(85.7)	
N1		0	(0)	2	(14.3)	
Treatment	No (%)					<0.01
ESBS		13	(92.9)	2	(14.3)	
Craniotomy combined with ESBS		1	(7.1)	11	(78.6)	
CRT		0	(0)	1	(7.1)	

ESBS, endoscopic skull base surgery; n.s., not significant.

In the last two decades, ESBS has been introduced for the resection of ONBs (9, 28). Previous reports indicate that ONBs resectable by ESBS have a good prognosis (29–31). Hanna et al. reported that ESBS had favorable outcomes in well-selected patients with appropriate adjuvant therapy (10). Further, ESBS is less invasive as it requires neither a skin incision nor the retraction of the frontal lobe. Another advantage of ESBS is that the surgeon can resect the tumor in the nasal cavity and paranasal sinuses with an enlarged view as provided by transnasal endoscopy (32, 33). These advantages could lead to a favorable prognosis in cases in which the tumor is completely resected by ESBS (28, 34). We consider infiltration of the brain parenchyma and dura mater beyond the medial wall of the orbit and the posterior wall of the frontal sinus to be a contraindication for ESBS. In this study, the results of treatment in cases that could be resected endoscopically were comparable to those treated by craniotomy. Overall, the results of treatment at our hospital have been generally comparable to past case reports, and are treatment protocol considered to be acceptable.

One of the issues with ONB is that the staging system has not been updated. To date, two staging systems, the modified Kadish staging system (35) and the Dulguerov staging system (36), have frequently been used for ONB. Despite the widespread recognition of ESBS, the staging systems for ONB have not been revised since the 1990s. The Kadish staging system was proposed in 1976, based on the retrospective analysis of 17 patients with ONB. In the original report, the patients were retrospectively classified into three groups for prognostic evaluation (37). The staging system was revised in 1993 by adding Kadish stage D, which corresponded to cases with distant metastasis (35). Meanwhile, Dulguerov proposed a new staging system in 1992 (36) based on the retrospective data from 26 cases with ONB at a single center. Although both staging systems were proposed for prognostic evaluation, not for the selection of treatment strategies, these have been widely used not only for the evaluation of ONB but also for the selection of treatment strategies for over 30 years (38). Choby et al. have proposed a new staging system incorporating Hyam grade and report that it is useful in predicting prognosis, but it is a new concept that needs further validation (39). While some cases classified as modified Kadish stage C can be resected and the dura matter reconstructed with ESBS alone, other patients with extensive modified Kadish stage C tumors require a craniotomy. Moreover, some cases classified as T4 disease under the Dulguerov staging system, where the tumor involves the dura matter at least in part, can be treated by endoscopic resection, depending on the extent of the lesion involvement in the dura matter. In the endoscopic era, the criteria adopted under the modified Kadish and Dulguerov staging systems have not contributed to the selection of the appropriate surgical approach. As mentioned in the previous section, patients who can be resected endoscopically have a better prognosis and fewer complications, and it is expected that a new staging system for ONB that considers endoscopic resection will be developed in the future.

Previous reports have shown that ESBS is considered less invasive than craniectomy and has been reported to have a lower complication rate (28), but few reports have examined the perioperative period in detail (40). As for complications in our institution, CSF leakage, deep vein thrombosis, and frontal bone necrosis were observed only in patients undergoing craniotomy, while there were no complications requiring treatment in the patients receiving ESBS. Severe complications were significantly less common in ESBS. As ESBS is less invasive, not requiring a skin incision or retraction of the frontal lobe, it is expected to reduce operative time and allow early weaning. Although there have been reports of shorter hospitalization (41), there have been few detailed reports on the perioperative period. In this study, the patients receiving ESBS demonstrated a significantly shorter operating time, period from operation to ambulation, hospitalization period, and less blood loss.

This study has some limitations. Due to the rarity of ONB, the sample size was relatively small. In addition, this is a retrospective study where the decision to perform ESBS alone or combined with craniotomy had been already made prior to surgery. A prospective study enrolling a more significant number of patients with ONB in multiple facilities is necessary to confirm the outcomes of the treatment protocol and its low complication rate.

Recently unilateral resection of ONB has been performed in some cases for smell preservation (42). In our institution, unilateral resection of ONB was started in 2018, and only one case with unilateral resection is included in this report. Therefore, the number of cases and the observation period were insufficient to incorporate unilateral resection. However, it is necessary to consider the indications for unilateral resection in the future.

## Conclusion

Our treatment protocol for ONB was found to be useful with acceptable oncological outcomes. Patients who underwent endoscopic resection had lower complication rates and better perioperative findings than those who underwent craniotomy. With appropriate case selection, ESBS is considered a useful approach for ONB.

## Data availability statement

The raw data supporting the conclusions of this article will be made available by the authors, without undue reservation.

## Ethics statement

The studies involving humans were approved by The Institutional Review Board for Clinical Research of Hokkaido University Hospital, Sapporo, Japan (019-0242). The studies were conducted in accordance with the local legislation and institutional requirements. Written informed consent for participation in this study was provided by the participants’ legal guardians/next of kin.

## Author contributions

AN: Conceptualization, Data curation, Formal analysis, Funding acquisition, Investigation, Methodology, Project administration, Resources, Software, Validation, Visualization, Writing – original draft, Writing – review & editing. HM: Conceptualization, Data curation, Investigation, Supervision, Validation, Writing – review & editing. MS: Conceptualization, Formal analysis, Project administration, Supervision, Validation, Visualization, Writing – review & editing. YN: Methodology, Conceptualization, Project administration, Validation, Writing – review & editing. SY: Supervision, Validation, Writing – review & editing. YI: Supervision, Validation, Writing – review & editing. SKa: Supervision, Validation, Writing – review & editing. NT: Supervision, Validation, Writing – review & editing. AyH: Supervision, Validation, Writing – review & editing. TS: Supervision, Validation, Writing – review & editing. SKi: Validation, Writing – review & editing. SH: Validation, Writing – review & editing. JT: Validation, Writing – review & editing. YS: Supervision, Writing – review & editing. TM: Validation, Writing – review & editing. KY: Validation, Writing – review & editing. HA: Supervision, Validation, Writing – review & editing. IK: Supervision, Validation, Writing – review & editing. MF: Supervision, Validation, Writing – review & editing. AkH: Conceptualization, Project administration, Supervision, Validation, Writing – review & editing.
